# The Clinical Significance of Glycoprotein Phospholipase D Levels in Distinguishing Early Stage Latent Autoimmune Diabetes in Adults and Type 2 Diabetes

**DOI:** 10.1371/journal.pone.0156959

**Published:** 2016-06-28

**Authors:** Wen Qin, Yu-Zhen Liang, Bao-Yu Qin, Jia-Li Zhang, Ning Xia

**Affiliations:** 1 Department of pathology, The First Affiliated Hospital of Guangxi Medical University, Nanning, Guangxi Province, China; 2 Department of Endocrinology and Metabolism, The First Affiliated Hospital of Guangxi Medical University, Nanning, Guangxi Province, China; 3 Department of Elderly Endocrinology, The First Affiliated Hospital of Guangxi Medical University, Nanning, Guangxi Province, China; West China Second Hospital, Sichuan University, CHINA

## Abstract

Autoantibodies have been widely used as markers of latent autoimmune diabetes in adults (LADA); however, the specificity and sensitivity of autoantibodies as markers of LADA are weak compared with those found in type 1 diabetes (T1DM). In this study, we aimed to identify other plasma proteins as potential candidates that can be used effectively to determine early stage LADA and type 2 diabetes (T2DM) to facilitate early diagnosis and treatment. These issues were addressed by studying new-onset ‘classic’ T1DM (n = 156), LADA (n = 174), T2DM (n = 195) and healthy cohorts (n = 166). Plasma samples were obtained from the four cohorts. We employed isobaric tag for relative and absolute quantitation (iTRAQ) together with liquid chromatography tandem mass spectrometry (LC-MS) to identify plasma proteins with significant changes in LADA. The changes were validated by Western blot and ELISA analyses. Among the four cohorts, 311 unique proteins were identified in three iTRAQ runs, with 157 present across the three data sets. Among them, 49/311 (16.0%) proteins had significant changes in LADA compared with normal controls, including glycoprotein phospholipase D (GPLD1), which was upregulated in LADA. Western blot and ELISA analyses showed that GPLD1 levels were higher in both LADA and T1DM cohorts than in both T2DM and healthy cohorts, while there were no significant differences in the plasma concentrations of GPLD1 between the LADA and T1DM cohorts. GPLD1 is implicated as a potential candidate plasma protein for determining early stage LADA and T2DM.

## Introduction

Latent autoimmune diabetes in adults (LADA) accounts for 12% of all cases of diabetes in epidemiological studies, with an incidence two or three times greater than that of ‘classic’ type 1 diabetes mellitus (T1DM) in the general population[1]. However, this disorder is not well-researched compared with classic T1DM and type 2 diabetes mellitus (T2DM)[2]. Since LADA shares a number of characteristics with classic T1DM, it is also known as latent T1DM or slowly progressive T1DM[3]. Furthermore, the treatment of LADA and T1DM is similar. However, due to the similarities in the clinical presentation of patients with T2DM or LADA in the early stages[4], many cases of LADA remain misclassified as T2DM. Furthermore, treatment requirements of patients with LADA may be different from those with T2DM[5] and their glycemic control is relatively poor[6]. Thus, distinguishing early stage LADA and T2DM is of great importance.

Currently, the diagnosis of LADA is based on the presence of four major circulating islet autoantibodies; glutamic acid decarboxylase autoantibodies (GADA), insulinoma-associated antigen-2 (IA-2A), insulin autoantibodies (IAA), and islet cell autoantibodies (ICA), which also exist in classic T1DM[7]. Among them, GADA is considered to be the most sensitive and specific biomarker of LADA[8]. Although the positive rate and titer of autoantibodies could be useful for risk stratification and accurate therapeutic choice in LADA[9], both parameters are relatively low in LADA patients compared with those in classic T1DM[10]. Furthermore, between 2% and 5% of patients with autoimmune diabetes (T1DM and LADA) are negative for the four classic antibodies (GADA, IA-2A, IAA, and ICA). Therefore, the search for novel candidate autoantibodies continues[11]. Although novel autoantibodies, such as those specific for zinc transporter 8 (ZnT8A)[12], have been identified, these potential biomarkers do not exhibit superior specificity and sensitivity in the detection of LADA. Thus, the identification of a new candidate plasma protein that can be used effectively to distinguish early stage LADA and T2DM is of great importance.

In this study, we employed iTRAQ proteomics, Western blotting, and ELISA to investigate the plasma of patients with LADA, classic T1DM, T2DM, and healthy adults, with the aim of identifying an ideal candidate plasma protein with the advantages of sensitivity and specificity in detecting LADA in the early stages.

## Materials and Methods

### Participants

A total of 174 LADA patients, 156 classic T1DM patients, 195 T2DM patients, and 166 healthy adults were enrolled as four cohorts in the study at the First Affiliated Hospital of Guangxi Medical University (China) from October 2011 to July 2014. LADA was diagnosed if: (1) the age at onset was >35 y; (2) At least one of four major circulating islet autoantibodies (GADA, ICA, IAA, IA-2A) was positive; and (3) insulin treatment was not started in the first 6 months after diagnosis. Individuals with T2DM were autoantibody-negative and insulin-independent at diagnosis. Patients with classic T1DM were autoantibody-positive and required prompt insulin therapy at diagnosis. The disease cohorts had been diagnosed with diabetes for ≤6 months and without diabetic complications. All patients were of Asian ancestry. The following exclusion criteria were applied: (1) Maternally Inherited Diabetes and Deafness (MIDD); (2) maturity-onset diabetes of the young (MODY); (3) gestational diabetes mellitus (GDM); and (4) other autoimmune diseases. The clinical characteristics of the participants are shown in Table 1, S1, S2, S3 and S4 Tables.

**Table 1:** The clinical characteristics of the participants.

	N	Age (years)	Sex (male/female)	Diabetes duration (months)	HbA1c (%)	Glucose, fasting (mmol/l)	Glucose, non-fasting (mmol/l)	C-peptide, fasting (ng/ml)	C-peptide, non-fasting (ng/ml)	GADA(+) or ICA(+) or IAA(+) or IA-2A(+)
LADA	174	37.2±5.0	96/78	4.8±1.1	8.2±0.8	9.2±1.7	15.8±1.5	0.7±0.1	1.3±0.2	174
T1DM	156	12.2±3.8^a^	84/72	4.5±1.2	8.6±0.8^a^	10.0±1.6^a^	17.0±1.9^a^	0.5±0.1^a^	1.0±0.2^a^	156
T2DM	195	38.2±4.7	111/84	4.7±1.2	7.8±0.7^a^	9.0±1.2	13.8±0.8^a^	2.0±0.5^a^	6.0±1.5^a^	0
NC	166	36.8±4.6	96/70	-------	5.1±0.4^a^	5.1±0.7^a^	6.1±1.1^a^	1.6±0.6^a^	5.6±1.9^a^	0

Data represent the number of cases or $\bar{x}\pm s$ or *M*; **P < 0.01;

^a^compared with LADA, *P* < 0.05

Glucose, non-fasting: glucose 2 hours after a meal; C-peptide, non-fasting: C-peptide 2 hours after a meal

### Ethics Statement

This study was approved by our Institutional Review Board (NO. EHBHKY 2011-KY-134), and informed patient consent was obtained before study participation according to institutional and native guidelines.

### Specimen Collection

Blood samples were collected from the four groups after an overnight fast. Only newly diagnosed patients were involved in this study and blood samples were collected without medication. Blood was collected from each participant in K2 EDTA-coated tubes and centrifuged within 30 min of collection at 3,000 ×*g* for 5 min at 4°C. Aliquots of the plasma layer were then stored at −80°C.

Biochemical indexes such as GADA, ICA, IAA, IA-2A, fasting C-peptide levels, non-fasting C-peptide levels, fasting plasma glucose levels, non-fasting plasma glucose levels, and HbA1c were determined in each group. GADA, ICA, IAA and IA-2A levels was measured by ELISA. Samples selected for three iTRAQ runs were obtained from patients (matched for sex and biochemical indexes) with classic T1DM (n = 30), LADA (n = 30), T2DM (n = 30), and healthy adult controls (n = 30). Three pooled samples were created prior to immunodepletion of high-abundance proteins by the accumulation of 15 μl of each plasma sample from 10 patients in each group.

### Sample Preparation for Mass Spectrometry Analysis

#### Removal of high-abundance proteins

Potential candidate plasma protein for determining LADA and T2DM require enrichment as their heterogeneity and low abundance make them difficult to analyze from among a complex mixture. Enrichment strategies for low-abundance plasma proteins usually rely on immunodepletion of high-abundance proteins[13, 14]. In the present study the high-abundance proteins among three pooled samples were removed by human 14 multiple affinity removal system columns (Agilent Technologies, Palo Alto, CA, USA)[15]. The samples were then desalted and concentrated at 4°C by ultrafiltration using 3-kDa-cut-off membranes (Millipore, Barueri, Sao Paulo, Brazil). Sample concentrations were estimated by the Bradford method and then aliquoted (100 μg protein) into 1 ml centrifuge tubes for vacuum freeze-drying.

#### Protein digestion and iTRAQ labeling

According to the standard protocol supplied by the manufacturer (Applied Biosystems, Foster City, CA, USA), 100 μg of freeze-dried protein samples were redissolved, denatured, reduced, the cysteines blocked, and digested. Each iTRAQ reagent (Applied Biosystems) was then dissolved in 50 μl isopropanol, vortexed and added to each sample pool for incubation (≥2 h at room temperature). Isobaric tags, 114, 116, 117, and 118 m/z, were added to the T2DM, LADA, classic type 1 diabetes, and healthy adults sample pool, respectively, according to the manufacturer’s instructions. Samples were then mixed in equal ratios and dried in a centrifugal vacuum concentrator to remove isopropanol.

#### Strong cation exchange fractionation

Following desalination by C18 Micro spin columns (Ultra Micro Spin Column 2–100 μl) and vacuum drying at low temperature, the samples were fractionated off-line on a strong cation exchange (SCX) column (PolyLC, Columbia, USA). Redissolved samples were added and peptides were then eluted stepwise with solutions of increasing concentration of KCl (200 mM, 400 mM, and 500 mM) in 10 mM KH_2_PO_4_, 25% acetonitrile, pH 3.0. Fractions were collected every 2 to 5 min and combined into 21 fractions depending on the intensity of UV absorbance at 214 nm. Fractions were dried by vacuum centrifugation.

### LC-MS/MS Analysis

All strong cation exchange (SCX) was performed under the following conditions: column size = 150 × 2.1 mm, 5 μm, 200 Å, flow rate = 0.2 ml/min, PolySulfethyl A; PolyLC. Fractions were analyzed on a Tempo^™^ LC-MALDI spotting system (Applied Biosystems). Peptides were separated at a flow rate of 2 μl/min, eluted with a 90 min gradient from 8%–40% mobile phase B (98% acetonitrile, 0.1% trifluoroacetic acid) and monitored by UV absorbance at 214 nm. Peptide-containing LC spots were submitted to a 5800 MALDI TOF/TOF^™^ analyzer (Applied Biosystems). MS full-scan spectra were acquired from 800–4,000 m/z. Data-dependent tandem MS settings included acquisition of up to 20 of the most intense ion signals per spot. Raw data processing, protein identification, protein relative quantitation and statistical analyses were undertaken with ProteinPilot Software Version 4.0 (Applied Biosystems) against the UniProt database. Protein confidence was set at 95% (equivalent to Unused ProtScore of 1.3). Proteins were accepted with a false discovery rate (FDR) of 1%. Only the proteins identified from three iTRAQ runs with *P*-values <0.05 (compared with the LADA cohort) and at least two peptides were accepted as unique. Only proteins with a relative expression ratio of ≤0.8- or ≥1.2 between two groups were accepted as significantly down- or upregulated[16, 17].

### Functional Annotation of Proteins

To select plasma proteins as potential candidates for early diagnosis of LADA, we used the web tools provided by the DAVID (http://david.abcc.ncifcrf.gov/), UniProt (http://www.uniprot.org/), and KEGG databases (http://www.genome.jp/kegg/) to search for functional annotation terms (FATs) and pathways that are enriched among the identified differentially expressed proteins.

### Western Blot Analysis

Aliquots of plasma were fractionated by SDS-polyacrylamide gel electrophoresis and transferred to PVDF membranes (0.2 μm; Millipore). After blocking in 5% non-fat milk at room temperature for 2 h and washing with PBST (3×5 min), membranes were incubated with the primary detection antibody (ab51356, mouse-anti-human glycoprotein phospholipase D monoclonal antibody (GPLD1), Abcam, Cambridge, UK) diluted 1:500. Membranes were washed three times in PBST before being incubated with IRDye 800CW secondary antibodies (926–32210, goat (polyclonal) anti-mouse IgG (H+L), 1:5,000, LI-COR Biosciences, Lincoln, USA) in blocking buffer for 2 h. Detection was performed with the Odyssey imaging system (LI-COR Biosciences, Lincoln, USA). GPLD1 isolated from human plasma was used as a positive control. C3b-α was used as a loading control.

### ELISA

Plasma GPLD1 concentrations were assayed using an anti-human GPLD1 ELISA kit (USCN Life Sciences, Wuhang, China) in accordance with the manufacturer’s instructions. The absorbance was read at 450 nm on a microplate reader (Bio-Rad, Hercules, USA).

### Statistical Analysis

Statistical analysis was performed using SPSS 19.0 statistical software. Normal distribution was confirmed for all variables using the Kolmogorov-Smirnov test. Normally distributed data were expressed as the mean ± SD. The SNK-q test was used for comparisons between multiple groups. The attribute data were analyzed by chi-square test. Receiver operating characteristic (ROC) curve analysis was used to assess the discriminating power of plasma GPLD1 levels to detect LADA. *P*-values < 0.05 were considered to indicate statistical significance.

## Results

**Fig 1:**
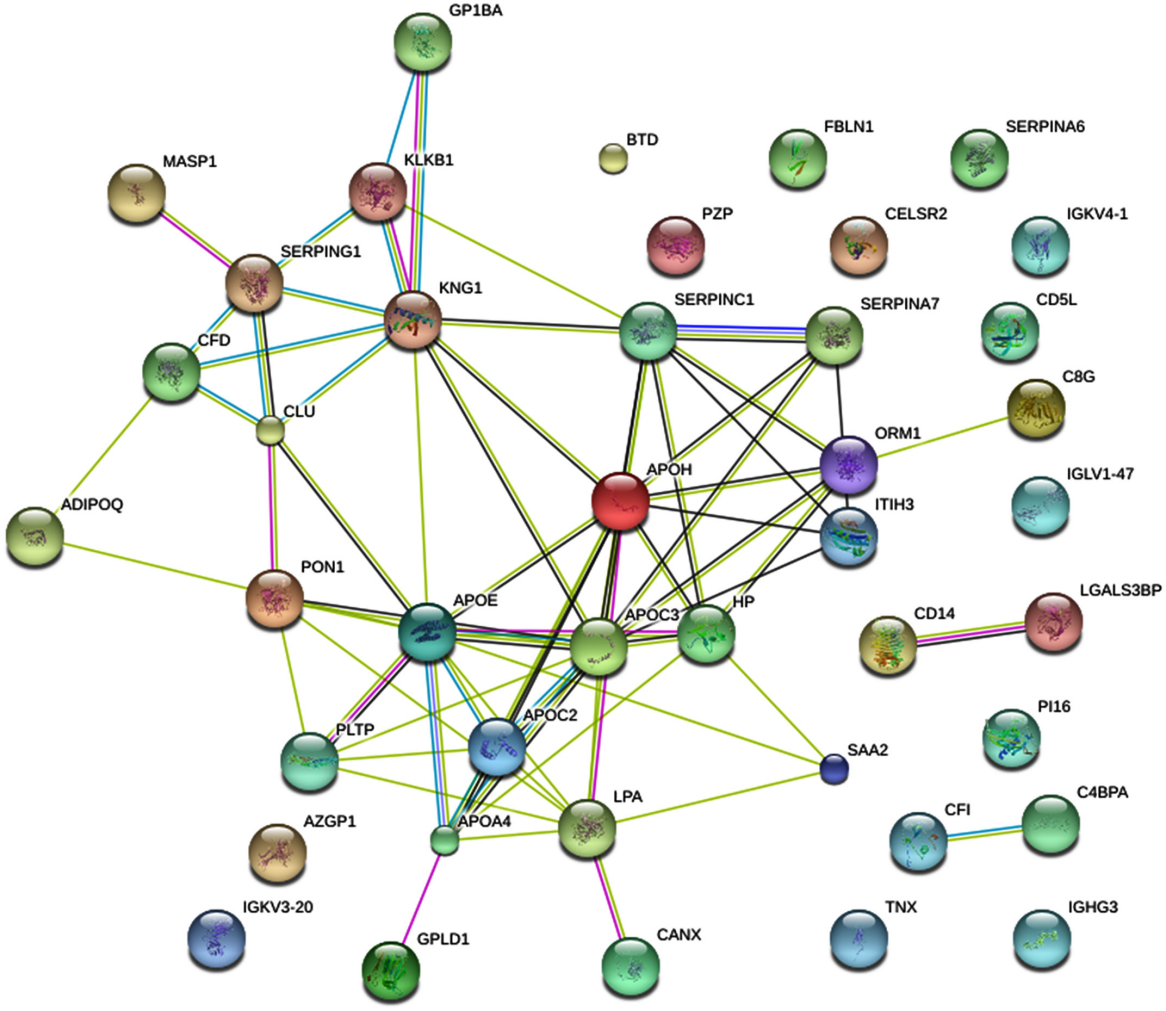
Protein-protein interactions among differentially expressed proteins predicted by ‘String’. Protein-protein interaction The interactions include direct (physical) and indirect (functional) associations and are derived from four sources: genomic context, high-throughput experiments, (conserved) coexpression, and previous knowledge. GPLD1 associates with the most intense protein interaction network group.

This study identified and quantified 311 plasma proteins in three iTRAQ runs; 157 of them were present across the three data sets in the LADA and normal control groups. Statistical analysis revealed 49 proteins with significant changes in abundance in the LADA group compared with the normal control group (Table 2). Fig 1 shows the interactions among the 49 proteins. Candidate proteins were selected for further validation studies based on the following criteria: (1) the candidate proteins should not be among the 14 high-abundance proteins that were removed by the Agilent human 14 multiple affinity removal system columns; (2) potential biological relevance to diabetes determined by literature reviews; and (3) raw concentration of protein in plasma higher than 2 fm/L (equivalent to approximately 0.1 ng of a 50 kDa protein), which is within the range of Western blot detection limits. According to these criteria and bioinformatics analysis (Table 3), subsequent studies were focused on GPLD1, APOC3, CD14, KLKB1, and SERPING1, which exhibited differential expression in both the LADA and T1DM cohorts when compared with the T2DM and normal control cohorts. Although the results indicated that CD14 participates in the mechanisms of a large number of human diseases, this marker lacked specificity. It has been reported that APOC3 is involved in type 2 diabetes. KLKB1and SERPING1 are involved in complement and coagulation cascades. Thus, these markers were not considered to be relevant to the development of LADA and T2DM, leaving GPLD1 as the only potential biomarker.

**Table 2:** Results of iTRAQ for 49 proteins with significant changes in LADA compared with controls.

	Accession	Name	114:116 (T2DM:LADA)	PVal 114:116 (PVal T2DM:LADA)	117:116 (T1DM:LADA)	PVal 117:116 (PVal T1DM:LADA)	118:116 (Normal:LADA)	PVal 118:116 (PVal normal:LADA)
1	P01008	ANT3	0.1995	0.0000	0.7798	0.0412	0.5297	0.0105
2	P06727	APOA4	0.8941	0.0983	0.7987	0.0460	0.7037	0.0407
3	P01042	KNG1	0.8628	0.1276	0.9769	0.4192	0.7796	0.0453
4	P25311	ZA2G	0.4365	0.0095	0.5445	0.0238	0.7112	0.0481
5	P10909	CLUS	0.1995	0.0000	0.9204	0.4239	0.6982	0.0368
6	P03952	KLKB1	0.6207	0.0234	0.9376	0.4525	0.4043	0.0093
7	P05155	SERPING1	0.5754	0.0140	0.9908	0.4615	0.2871	0.0064
8	Q06033	ITIH3	0.7047	0.0401	1.0765	0.4720	0.7379	0.0409
9	P08185	CBG	0.6026	0.0215	1.1482	0.2728	0.7047	0.0432
10	P05543	THBG	0.7047	0.0401	1.0375	0.4325	0.7311	0.0440
11	P27169	PON1	0.1941	0.0000	0.7656	0.0426	0.6792	0.0324
12	P23142	FBLN1	0.7244	0.0423	1.2134	0.0497	0.6668	0.0391
13	P08519	APOA	0.9833	0.1320	0.7768	0.0438	0.5792	0.0236
14	P80108	GPLD1	0.5208	0.0194	0.9503	0.4562	0.5301	0.0125
15	P01880-2	IGHD	1.0264	0.2310	0.6090	0.0397	0.4132	0.0099
16	A8K660	A8K660	0.8381	0.0952	1.1264	0.4631	0.7305	0.0420
17	P48740	MASP1	0.6668	0.0285	0.8318	0.1800	0.6252	0.0317
18	P55058	PLTP	0.7311	0.0476	1.2246	0.0438	0.5297	0.0105
19	Q15848	ADIPO	0.7870	0.0489	1.0765	0.4562	0.7656	0.0406
20	P22105	TENX	0.6792	0.0271	0.9817	0.4678	0.7586	0.0412
21	P01717	LV403	0.8880	0.0800	0.5970	0.0152	0.7964	0.0489
22	P27824	CALX	0.7311	0.0412	0.8790	0.0472	0.5916	0.0288
23	P00738	HPT	1.6904	0.0008	1.7865	0.0092	3.3729	0.0000
24	P02749	APOH	0.9198	0.1478	0.7917	0.0486	1.2196	0.0476
25	P20742	PZP	1.5091	0.0019	0.9166	0.4178	1.2047	0.0491
26	P02649	APOE	2.8840	0.0000	2.5119	0.0000	2.1677	0.0000
27	P01860	IGHG3	1.0848	0.2820	0.7549	0.0493	1.8542	0.0009
28	Q5VVQ8	Q5VVQ8	0.8821	0.0793	1.0055	0.6210	1.3084	0.0253
29	D9YZU5	D9YZU5	0.9760	0.3361	1.0594	0.6210	1.2446	0.0400
30	P02656	APOC3	2.2909	0.0000	1.4859	0.0078	1.7701	0.0052
31	P02763	A1AG1	1.6881	0.0009	1.1056	0.6170	1.5502	0.0081
32	P05156	CFAI	0.9817	0.1528	0.9640	0.6531	1.2131	0.0476
33	P43251	BTD	1.2738	0.0329	0.9246	0.5177	1.2376	0.0476
34	P07360	CO8G	0.9001	0.1457	0.9860	0.5346	1.2461	0.0391
35	P02655	APOC2	1.5364	0.0013	1.3317	0.0146	1.2047	0.0480
36	D1MGQ2	D1MGQ2	1.0106	0.1893	1.0987	0.5199	1.2150	0.0474
37	P02735	SAA	1.4588	0.0069	0.3048	0.0000	6.7920	0.0000
38	P18136	KV313	1.4060	0.0053	0.7379	0.0467	1.4454	0.0179
39	P08571	CD14	2.1878	0.0000	1.3305	0.0260	1.5276	0.0083
40	P01623	KV305	1.1847	0.0900	0.9273	0.4274	1.2100	0.0455
41	Q08380	LG3BP	1.2474	0.0432	1.1803	0.2140	1.3552	0.0274
42	P06314	KV404	1.1144	0.1537	1.0583	0.5380	1.3027	0.0365
43	O43866	CD5L	0.8720	0.0752	0.9814	0.4150	1.4306	0.0210
44	B4DVE1	B4DVE1	1.2646	0.0455	1.1683	0.3147	1.2182	0.0401
45	Q6UXB8	PI16	2.7040	0.0000	0.8630	0.2145	6.0813	0.0000
46	P04208	LV106	1.4859	0.0107	1.1912	0.0657	1.2942	0.0396
47	P00746	CFAD	2.2909	0.0000	0.8166	0.2769	2.1878	0.0000
48	Q9HCU4	CELR2	12.2462	0.0000	5.2000	0.0000	7.2444	0.0000
49	P07359	GP1BA	20.1372	0.0000	21.6770	0.0000	9.4624	0.0000

**Table 3:** Significant signaling pathway and proteins implicated in LADA.

	Pathway	Protein
hsa03320	PPAR signaling pathway	APOC3
hsa04610	Complement and coagulation cascades	KLKB1, SERPING1
hsa04010	MAPK signaling pathway	CD14
hsa04620	Toll-like receptor signaling pathway	CD14
hsa04930	Type II diabetes mellitus	APOC3
hsa04145	Phagosome	CD14
hsa00563	Glycosylphosphatidylinositol (GPI)-anchor biosynthesis	GPLD1

### Candidate Verification by Western Blot Analysis

**Fig 2:**
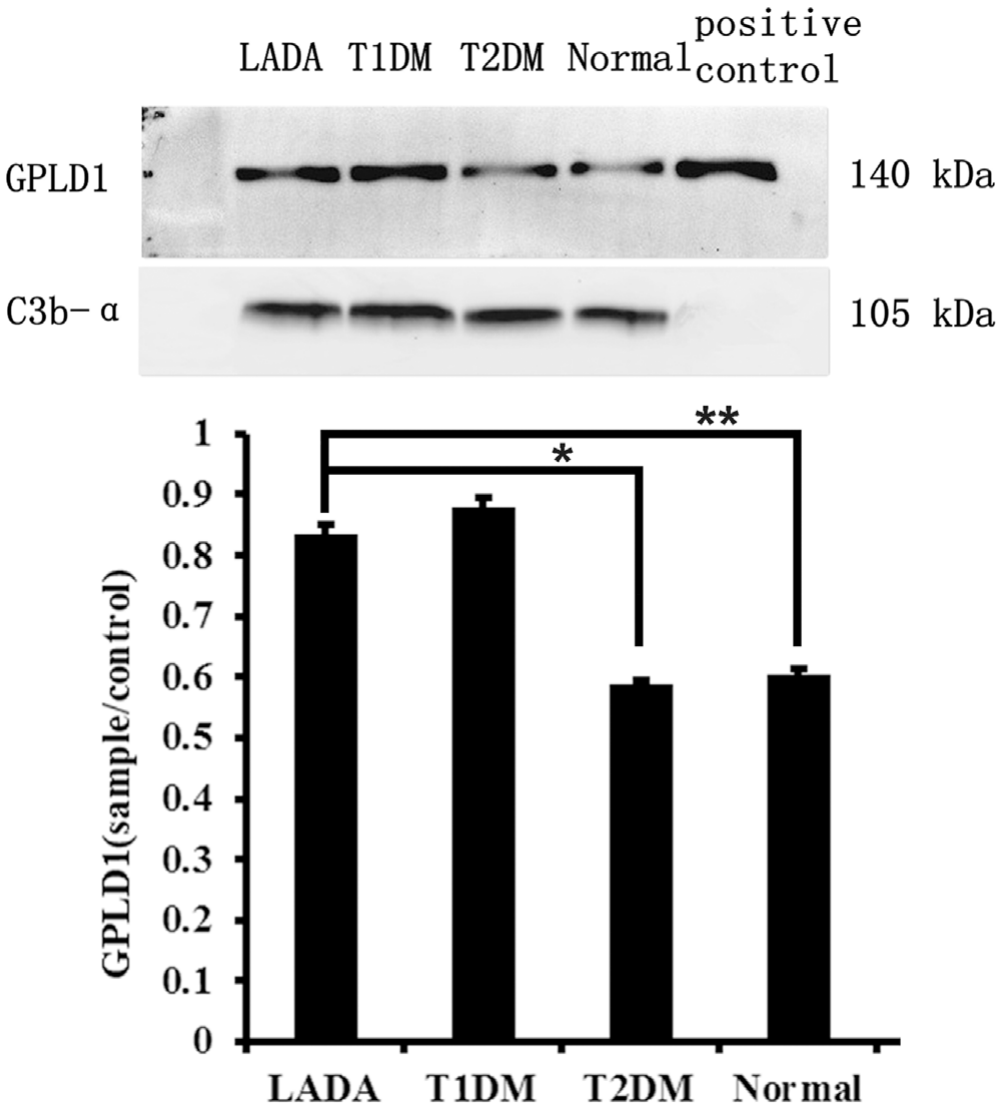
Western blot analysis of the relative expression of GPLD1 among the four cohorts. GPLD1 isolated from human plasma was used as a positive control. No significant difference in GPLD1 expression was observed between the LADA and ‘classic’ type 1 diabetes cohorts (*P* >0.05); however, there were significant differences between the LADA cohort and the type 2 diabetes and normal control cohorts (*P*<0.05).

GPLD1 was selected for further investigation based on the results of the iTRAQ and LC-MS analyses. Western blot analysis was used to further confirm the changes in plasma GPLD1 levels. The predicted band-size of GPLD1 is 92 kDa; however, the circulating GPLD1 protein is approximately 140 kDa due to post-translational modification. A band of approximately 140 kDa was detected by Western blot in each plasma sample. Different levels of GPLD1 expression were observed in each group. All experiments were performed in triplicate and GPLD1 isolated from human plasma was used as a positive control. Western blot analysis showed that the relative expression of GPLD1 among the four cohorts [LADA (n = 174), classic type 1 diabetes (n = 156), T2DM (n = 195) and normal controls (n = 166)] was 0.84±0.02, 0.88±0.02, 0.59±0.01, and 0.60±0.01, respectively. No significant differences were observed between the LADA and classic T1DM cohorts (*P >* 0.05). However, GPLD1 levels were significantly higher in the LADA cohort compared with those in the T2DM and normal control cohorts (*P* < 0.05). (Fig 2). These findings were consistent with the iTRAQ results.

### Candidate Verification by ELISA

**Fig 3:**
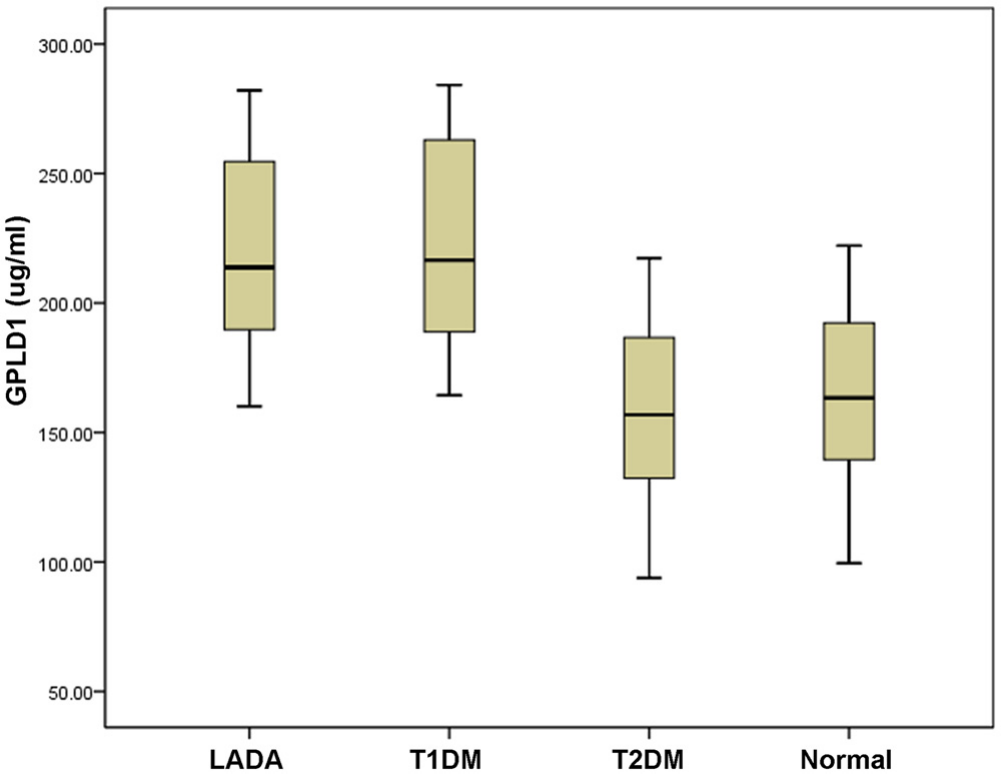
ELISA analysis of the concentration of GPLD1 among the four cohorts. No significant difference in GPLD1 expression was observed between the LADA and ‘classic’ type 1 diabetes cohorts (*P* >0.05); however, there were significant differences between the LADA cohort and the type 2 diabetes and normal control cohorts (*P*<0.05).

The ELISA results provided a curvilinear plot covering a range of 0.78–50 ng/ml. GPLD1 concentration was found to be highly correlated with the optical density (correlation coefficient, 0.9991). GPLD1 concentrations in the four cohorts [LADA (n = 174), classic T1DM (n = 156), T2DM (n = 195) and normal controls (n = 166)] were 218.09±35.56 μg/ml, 222.67±38.62 μg/ml, 155.85±37.94 μg/ml, and 162.88±34.66 μg/ml, respectively. There was no significant difference between the concentrations in the LADA and classic T1DM cohorts (*P* > 0.05). However, GPLD1 levels were significantly higher in the LADA cohort compared with those in the T2DM and normal control cohorts (*P* < 0.05). The results of the ELISA and Western blot analyses were comparable and consistent with our proteomics data, demonstrating significant GPLD1 upregulation in the LADA cohort (Fig 3).

### A Comparison of the Diagnostic Value of GPLD1 and GADA in LADA

**Fig 4:**
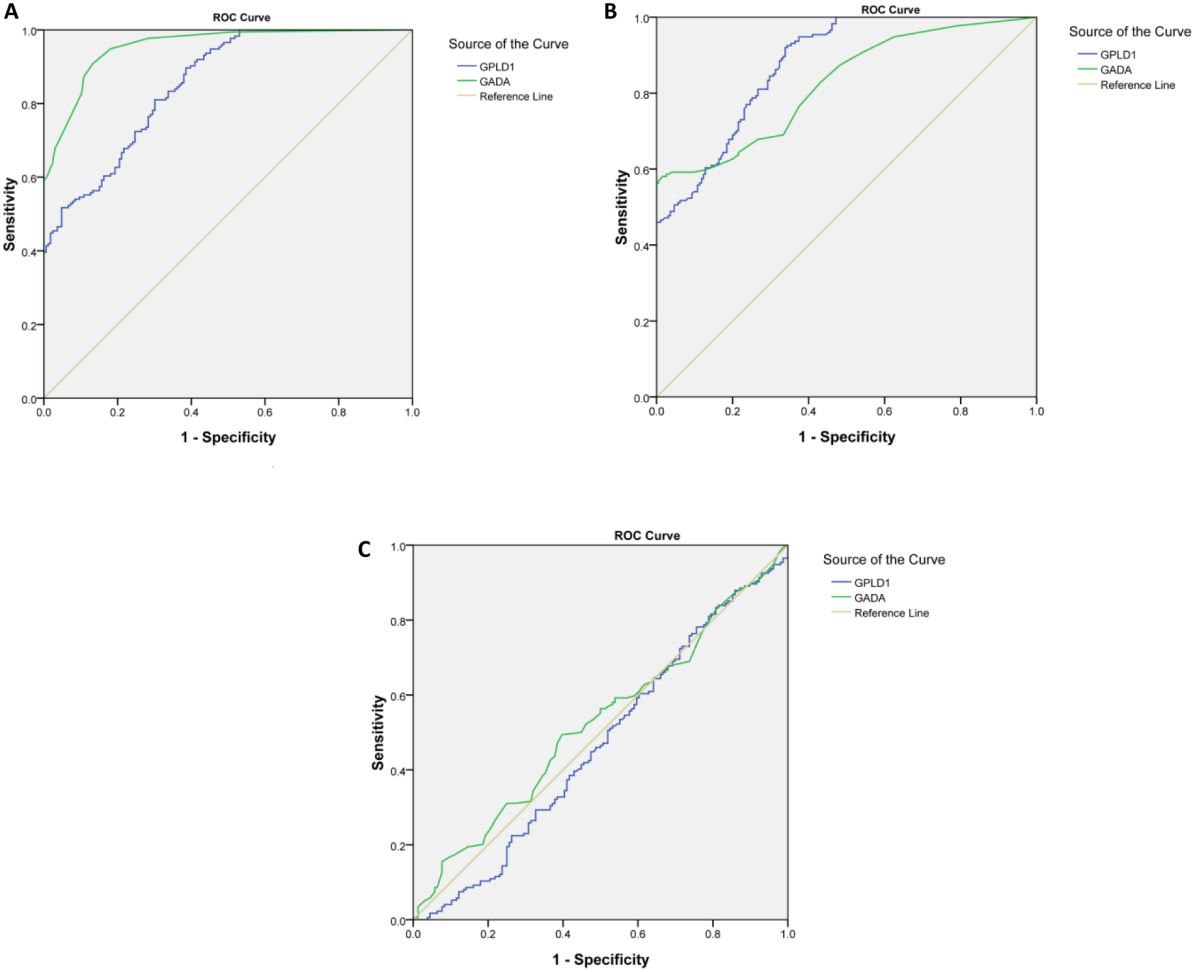
A comparison of the diagnostic value of GPLD1 and GADA in LADA. **(A) ROC curve obtained in LADA and healthy cohorts.** (B) ROC curve obtained in LADA and T2DM cohorts. (C) ROC curve obtained in LADA and T1DM cohorts.

As shown in Fig 4 and Table 4, the ROC curve was obtained by testing the concentrations of GPLD1 and GADA in the LADA, T1DM, T2DM and healthy control cohorts.

**Table 4:** Results of the ROC curve.

	ROC curve obtained in LADA and healthy cohorts	ROC curve obtained in LADA and T2DM cohorts	ROC curve obtained in LADA and T1DM cohorts
GPLD1			
Cut-off	211.269	213.846	216.536
Sensitivity	51.7%	50.0%	46.6%
Specificity	95%	95%	50%
AUC	0.854 (CI 95% 0.816–0.892)	0.877(CI 95% 0.844–0.909)	0.468(CI 95% 0.405–0.531)
GADA			
Cut-off	0.08 (antibody titer)	0.21 (antibody titer)	0.33 (antibody titer)
Sensitivity	69.0%	59.2%	56.3%
Specificity	96%	96%	50%
AUC	0.955 (CI 95% 0.936–0.974)	0.824(CI 95% 0.781–0.866)	0.525(CI 95% 0.462–0.587)

## Discussion

Autoantibodies are frequently found during the preclinical period of T1DM[18] and LADA[19]. These disorders share similar autoantibodies and clinical management and are not difficult to distinguish due to differences in the age at onset of diabetes. However, it is difficult to distinguish early stage LADA and T2DM in adults due to the similarities in clinical features; therefore, many cases of early stage LADA remain misclassified as T2DM. Thus, identification of a plasma protein for more efficient distinction between early stage LADA and T2DM is of great importance.

In this study, we employed iTRAQ and LC-MS to identify proteins showing significant changes in expression in LADA. We identified 311 unique proteins in three iTRAQ runs, with 157 present across the three data sets in the LADA and normal control groups. Among them, 49/311 (16.0%) proteins showed significant changes, including GPLD1, which was upregulated in both the LADA and T1DM cohorts compared with the T2DM and normal control cohorts. These results were further validated by Western blot and ELISA analyses. For all cases of incipient diabetes (Table 1), GPLD1 distinguished T2DM and LADA in the early stage. Moreover, there were no significant differences in the plasma concentrations of GPLD1 between the LADA and T1DM cohorts, indicating that, unlike the four autoantibodies currently used as markers, GPLD1 can be used to detect LADA and T1DM with equal efficiency. It was shown that GPLD1 can be used to determine LADA and T2DM cohorts, LADA and healthy cohorts with high diagnostic value in the ROC curve. However, the sensitivity and specificity of GADA was slightly higher than that of GPLD1 (Table 4). Our investigation of the significance of GPLD1 showed that the changes in the expression of GPLD1 and GADA was not always consistent. For example, some cases of LADA or T1DM tested positive for GADA but negative for GPLD1, while the reverse was true in some other cases. Thus, GPLD1 may be useful for diagnosis in some cases.

Previous research has shown that GPLD1 mRNA and protein levels are increased in mice that develop insulin-dependent T1DM spontaneously[20, 21]. However, the etiology of preclinical LADA[19] and the mechanisms underlying the changes in plasma concentrations of GPLD1 remain to be elucidated.

GPLD1 is a mammalian plasma protein (110–120 kDa). Liver and pancreatic islets are two likely sources of GPLD1 and GPLD1 cDNA has been isolated from both organs in multiple species. However, studies have demonstrated that brain, kidney, muscle, immunocytes, and inflammatory cells may also be sources[22]. The expression of GPLD1 mRNA in liver is higher than that in other organs[20]. The *GPLD1* gene, which is located on chromosome 6 and consists of 25 exons and was previously designated as the pancreatic form (EMBL/GenBank/DDBJ accession number L11702), is the only *GPLD1* gene in humans[23]. *GLPD1* is linked to susceptibility to pancreatic and gastric malignancies[24] and genetic variations are known to influence plasma GPLD1 levels[25]. The close relationship between GPLD1 and pancreatic islets might be an important cause of the increased plasma concentrations of GPLD1 among patients with LADA or T1DM.

As a hydrolase in the glycosylphosphatidylinositol-anchor biosynthesis pathway, GPLD1 releases GPI-anchored membrane proteins by hydrolyzing the anchor before and after its attachment to proteins. The GPI-anchor is a post-translational modification that covalently links many proteins to membranes, occurring in a wide variety of eukaryotes from yeast to mammals. In mammals, the GPI-anchor attaches many functional proteins, such as enzymes, receptors, cell adhesion molecules and differentiation antigens to cellular membranes[26]. It has been shown that the increasing release of GPI-anchor-dependent membrane proteins is associated with breast carcinoma[27]. GPLD1 has also been implicated in the mechanism underlying the involvement of GPLD1 in carcinoembryonic antigen (CEA) release from human colon cancer cells[28, 29].

Consequently, the potential role of GPLD1 in LADA is a current concern. It is well-known that GAD65, a predominant form of GAD in the pancreas, is important in LADA and classic T1DM. Although previous studies have shown that LADA patients tend to be GAD antibody-positive, the mechanism responsible for the generation of these antibodies is largely unknown. Interestingly, GAD_65_ is a soluble cytosolic protein which can be anchored to the membranes[30] and released from the membrane through changes in enzyme activity. Moreover, the correlation in the timing between GPLD1 upregulation and the emergence of GADA highlights the potential relationship between these two events. Therefore, the ability of GPLD1 to cleave GAD_65_ or anti-GAD from the GPI-anchor leading to LADA represents an issue for further investigation. It can be speculated that the development of other autoantibodies found in LADA might be explained by the same mechanism. Furthermore, accumulating evidence supports the associations among autoimmune diseases, with autoimmune co-morbidity typically involving T1DM or autoimmune thyroid disease (AITD) and multiple sclerosis; inflammatory bowel disease (IBD), T1DM or AITD and rheumatoid arthritis; or T1DM and AITD[31]. Moreover, the strong association of other autoimmune diseases with LADA, such as AITD or autoimmune Addison’s disease[32], suggests that they share a common underlying mechanism, which might be similar to that described involving the function of GPLD1 in GPI-anchor hydrolysis and the release of GPI-anchored proteins leading to the development of autoantibodies.

In addition, the potential regulatory role of GPLD1 in chronic inflammatory reactions might be another mechanism underlying the development of LADA. Previous data suggests that chronic inflammation of pancreatic islets is connected with the pathogenesis of T1DM[33]. In T1DM, the components of the inflammatory responses that contribute to β-cell destruction include CD4^+^and CD8^+^ T-cells, macrophages, and natural killer (NK) cells. The potential relationship between islet cell autoimmunity and inflammatory markers may be similar in LADA. It is possible that by hydrolyzing the GPI anchors of some inflammatory membrane proteins and upregulating macrophage cytokine expression, GPLD1 may play an important role in inflammation and in the pathogenesis of LADA. Furthermore, GPLD1, localized mainly in the Golgi, endoplasmic reticula, and vesicles, may enter the MHC-I processing pathway, depending on specific genes related to IDDM[34]. Other reports have indicated that the relationship between plasma GPLD1 and insulin resistance is controversial[35].

## Conclusion

In summary, several possibilities may explain the relationship between GPLD1 and LADA, including the release of antigens or antibodies by GPI-anchor hydrolysis, leading to chronic inflammation as a result of entry into the MHC-I processing pathway. It is possible that GPLD1 is not only a candidate plasma protein in determining early stage LADA and T2DM, but also a critical factor involved in the pathogenesis of LADA. As a functional enzyme, GPLD1 provides a significant advantage as a candidate plasma protein marker of early stage LADA and T2DM. First, accumulating evidence suggests that disease progression does not occur in the absence of a set of functional enzymes. Second, enzymes are relatively stable in vivo under normal conditions, while in contrast, they exhibit changes in stability under pathological conditions. A variety of enzymes, including amylase, aminotransferase, lactic dehydrogenase, and creatine phosphokinase, have been used as biomarkers in clinical practice.

Our data are the first to indicate the correlation between GPLD1 and early stage LADA. Among the established diagnostic methods, GADA has higher sensitivity and specificity than GPLD1, although GPLD1 may still be a promising candidate plasma protein for distinguishing between early stage LADA and T2DM. The early diagnosis of LADA could be important in determining the most appropriate therapeutic choice in clinical practice. Our data strongly support the conclusion that proteomics is a feasible strategy for the identification of candidate plasma proteins for early diagnosis of LADA. In this study, we provide preliminary evidence demonstrating the potential of GPLD1 as a candidate plasma protein that can be used effectively to distinguish between early stage LADA and T2DM. However, mechanistic studies of the signaling pathways implicated in this process are required and the clinical utility of GPLD1 requires confirmation in a further multicenter study. In addition, this research was conducted in an Asian population and the results should be confirmed in other ethnicities.

## Supporting Information

S1 Table The clinical characteristics of 174 LADA patients. Biochemical indexes such as GPLD1, GADA, fasting C-peptide levels, non-fasting C-peptide levels, fasting plasma glucose levels, non-fasting plasma glucose levels, and HbA1c were determined in each patient.
(XLS)

S2 Table The clinical characteristics of 156 classic T1DM patients. Biochemical indexes such as GPLD1, GADA, fasting C-peptide levels, non-fasting C-peptide levels, fasting plasma glucose levels, non-fasting plasma glucose levels, and HbA1c were determined in each patient.
(XLS)

S3 Table The clinical characteristics of 195 T2DM patients. Biochemical indexes such as GPLD1, GADA, fasting C-peptide levels, non-fasting C-peptide levels, fasting plasma glucose levels, non-fasting plasma glucose levels, and HbA1c were determined in each patient.
(XLS)

S4 Table The clinical characteristics of 166 healthy adults. Biochemical indexes such as GPLD1, GADA, fasting C-peptide levels, non-fasting C-peptide levels, fasting plasma glucose levels, non-fasting plasma glucose levels, and HbA1c were determined in each patient.
(XLS)
